# *ZmNF-YB10*, a maize NF-Y transcription factor, positively regulates drought and salt stress response in *Arabidopsis thaliana*

**DOI:** 10.1080/21645698.2024.2438421

**Published:** 2024-12-24

**Authors:** Yimeng Wang, Peng Jiao, Chenyang Wu, Chunlai Wang, Ke Shi, Xiaoqi Gao, Shuyan Guan, Yiyong Ma

**Affiliations:** a College of Agronomy, Jilin Agricultural University, Changchun, China; b Joint International Research Laboratory of Modern Agricultural Technology, Ministry of Education, Jilin Agricultural University, Changchun, China

**Keywords:** Arabidopsis, drought stress, maize, salt stress, yeast heterologous expression

## Abstract

Maize (*Zea mays* L.) is a major food and feed crop and an important raw material for energy, chemicals, and livestock. The NF-Y family of transcription factors in maize plays a crucial role in the regulation of plant development and response to environmental stress. In this study, we successfully cloned and characterized the maize NF-Y transcription factor gene *ZmNF-YB10*. We used bioinformatics, quantitative fluorescence PCR, and other techniques to analyze the basic properties of the gene, its tissue expression specificity, and its role in response to drought, salt, and other stresses. The results indicated that the gene was 1209 base pairs (bp) in length, with a coding sequence (CDS) region of 618 bp, encoding a polypeptide composed of 205 amino acid residues. This polypeptide has a theoretical isoelectric point of 5.85 and features a conserved structural domain unique to the NF-Y family. Quantitative fluorescence PCR results demonstrated that the *ZmNF-YB10* gene was differentially upregulated under drought and salt stress treatments but exhibited a negatively regulated expression pattern under alkali and cold stress treatments. Transgenic *Arabidopsis thaliana* subjected to drought and salt stress in soil showed greener leaves than wild-type *A. thaliana*. In addition, the overexpression lines showed reduced levels of hydrogen peroxide (H_2_O_2_), superoxide (O^2-^), and malondialdehyde (MDA) and increased activities of peroxidase (POD), catalase (CAT), and superoxide dismutase (SOD). Western blot analysis revealed a distinct band at 21.8 kDa. Salt and drought tolerance analyses conducted in *E. coli* BL21 indicated a positive regulation. In yeast cells, *ZmNF-YB10* exhibited a biological function that enhances salt and drought tolerance. Protein interactions were observed among the *ZmNF-YB10*, *ZmNF-YC2*, and *ZmNF-YC4* genes. It is hypothesized that the *ZmNF-YB10, ZmNF-YC2*, and *ZmNF-YC4* genes may play a role in the response to abiotic stresses, such as drought and salt tolerance, in maize.

## Introduction

Maize (*Zea mays* L.) is an annual herbaceous plant belonging to the grass family and is one of the world’s major food crops.^1^ Compared with other food crops, maize has a very high demand and utilization rate, making it an indispensable raw material for animal husbandry, the light industry, and various other sectors. Abiotic stress caused by harsh environmental factors severely affects approximately 10% of global croplands, resulting in yield losses that exceed 50%. Osmotic stress due to drought and salinization of arable land is a critical factor limiting plant growth. Previous studies have demonstrated that maize growth, development, yield, and quality are affected to varying degrees, exhibiting a declining trend under prolonged water deficit and high salt osmotic stress.^2^ Therefore, breeding maize to improve its resistance to abiotic stress and increase its yield is urgently required.

Nuclear factor Y (NF-Y) is a transcription factor that is ubiquitously found in higher eukaryotes. They are also known as CCAAT-binding factors (CBFs) and hemoglobin-activating proteins (HAPs) owing to their specific binding to the CCAAT box.^3^ The NF-Y transcription factor is a conserved heterotrimer^4^ consisting of three distinct subfamilies: NF-YA (HAP2/CBF-B), NF-YB (HAP3/CBF-A), and NF-YC (HAP5/CBF-C).^5^ Among these, NF-YB and NF-YC subunits can form heterodimers in the cytoplasm. Once transferred to the nucleus, the NF-YB/NF-YC heterodimer binds to the NF-YA subfamily, resulting in the formation of the NF-Y complex.^6^ The NF-Y complex has been extensively studied in animal systems, revealing that highly conserved structural domains are closely associated with DNA-binding and protein-protein interactions mediated by NF-Y transcription factors. In contrast to yeast and mammals, in which each subunit is encoded by a single gene, plants possess multiple NF-Y-subunit genes.^7^ For example, 25 *NF-Y* genes have been identified in the melon genome,^8^ including six *CmNF-YA*, 11 *CmNF-YB*, and eight *CmNF-YC*. A total of 37 *NF-Y* genes (10 *NF-YA*, 11 *NF-YB*, and 14 *NF-YC*) are present in wheat.^9^ Fifty-one *NF-Y* genes (ShNF-Y) have been identified in sugarcane,^10^ including nine *NF-YA*, 18 *NF-YB*, and 24 *NF-YC* genes. Ten *HAP2*, 11 *HAP3*, and seven *HAP5* genes have been identified in the rice genome.^11^ Sixty-four NF-Y gene members have been identified in alfalfa,^12^ including 11 *MsNF-YAs*, 33 *MsNF-YBs*, and 20 *MsNF-YCs*. In addition, 50 *ZmNF-Y* genes (14 *ZmNF-YA*, 18 *ZmNF-YB*, and 18 *ZmNF-YC*) have been identified in maize. In plants, *NF-Y* genes exhibit various expression patterns at different developmental stages or in response to environmental stress. Therefore, a combination of different subunits facilitates the assembly of NF-Y complexes with diverse functions.

Numerous studies have confirmed that the NF-Y complex specifically binds to highly conserved CCAAT sequences in the promoter regions of its target genes^13^ and plays a crucial role in plant embryo development, seed maturation, and responses to abiotic stress. For instance, overexpression of *OsHAP2E*^14^ confers salt and drought tolerance in rice, whereas overexpression of *OsNF-YA7*^15^ enhances drought resistance in the same crop. Conversely, *OsNF-YC5*^13^ negatively regulates salt tolerance in rice and *OsNF-YA3*^16^ modulates plant growth and osmotic stress tolerance by interacting with SLR1 and SAPK9. In wheat, overexpression of *NF-YA10* increases salinity sensitivity, whereas in *Arabidopsis*, it increases drought tolerance.^17^ In addition, the transcription factor *TaNF-YB2*^18^ interacts with the chaperone protein *TaNF-YA7/C7* to improve drought tolerance in wheat. *TaNF-YA7-5B*^19^ confers drought tolerance to plants by regulating osmotic stress, whereas *TaNF-YB11* enhances drought tolerance by modulating osmotic solute accumulation and balancing reactive oxygen species (ROS) levels. Overexpression of Ginkgo *GbNF-YA6* in *Arabidopsis thaliana* improves survival rates at high temperatures^20^ and enhances heat tolerance by regulating heat shock factors or interacting with heat shock proteins. When overexpressed in transgenic tobacco plants, the citrus gene *CsNF-YA5*^21^ reduces hydrogen peroxide (H_2_O_2_) production under dehydrated conditions and improves growth and photosynthetic rates under both normal and drought stress conditions. *Arabidopsis ABF3* and *ABF4* function in conjunction with NF-YC to promote flowering by inducing SOC1 transcription under drought conditions.^22^ Furthermore, overexpression of *NF-YB2* and *NF-YB3* in *Arabidopsis* specifically enhances drought and heat stress tolerance, respectively,^23^ with each knockout mutant exhibiting unfavorable stress-sensitive phenotypes. Loss of function of *Arabidopsis NF-YC1* results in a salt-sensitive phenotype.^24^ Transgenic *Arabidopsis* plants overexpressing *NFYA5*^25^ exhibit increased resistance to drought stress. *PdNF-YB7*^26^enhances drought tolerance in *A. thaliana*, resulting in higher yields even under water deficit conditions. *OxPwNF-YB3*^27^ in *Arabidopsis* accelerates flowering and provides significant tolerance to salinity, drought, and osmotic stress in seedlings. *StNF-YC9*^28^ plays a crucial role in drought tolerance, as it increases the photosynthetic rate, superoxide dismutase (SOD) activity, and proline accumulation while reducing malondialdehyde (MDA) content in potato. *CmNF-YB8*^29^ influences drought tolerance by modifying leaf cuticle thickness and regulating epidermal stomatal movement through the expression of *CmCIPK6* and *CmSHN3*. *Cdt-NF-YC1*^30^ enhances drought and salinity tolerance in transgenic rice by regulating genes in both abscisic acid (ABA)-dependent and -independent pathways. In summary, NF-Y plays a vital role in plant drought and salt tolerance, making it an important target in breeding programs aimed at improving plant stress tolerance.

In maize, *ZmNF-YA3*^31^ enhances drought and high-temperature tolerance by binding to the promoter regions of *bHLH92*, FAMA, and jasmonate activator MYC4, respectively. *ZmNF-YB16*^32^ improves drought tolerance and yield by enhancing the photosynthetic and antioxidant capacities of maize. Overexpression of *ZmNF-YB2* (*ZmDREB2A*) enhances heat tolerance in transgenic plants,^33^ whereas *ZmNF-YA1* improves heat tolerance in maize by modulating the heat shock response.^34^ Both *ZmNF-YA1* and *ZmNF-YB16* regulate the growth, development, and drought tolerance of maize.^35^ Maize *ZmNF-YC12*^36^ is a key transcriptional activator that regulates drought resistance and resilience. Transgenic *A. thaliana* expressing maize *ZmNF-YB13* exhibits enhanced drought resistance. Maize *ZmNF-YC8*^37^ is highly homologous to the *NF-YC2* gene of *A. thaliana*, which plays a significant regulatory role in flowering. Studies on the function of NF-Y have primarily focused on *Arabidopsis*, rice, and other model crops. Despite the relatively large number of NF-Y subunit members in maize, the maize NF-Y gene family has been poorly studied and the underlying mechanisms of NF-Y genes remain largely unknown.

In this study, we combined pre-laboratory drought and saline transcriptome databases to identify significantly upregulated transcription factor *ZmNF-YB10* genes. *A. thaliana* was transformed using the *Agrobacterium*-mediated method to obtain transgenic plants. The biological functions of *ZmNF-YB10* in maize were investigated under drought and salt stress. Additionally, the interacting proteins of *ZmNF-YB10* were identified through yeast two-hybrid experiments, providing a theoretical basis and technical support for the development of new maize germplasm and the selection of improved varieties.

## Materials and Methods

### Plant Materials and Growth Conditions

Maize inbred line B73 served as the test material for this experiment. Seeds of the B73 inbred line were sown in a mixture of peat soil and vermiculite (2:1 ratio) and cultured under greenhouse conditions at 25°C with a photoperiod of 16 hours of light and 8 hours of darkness, and relative humidity of 65%. At the three-leaf stage, maize roots, stems, and leaves were frozen in liquid nitrogen to extract the total RNA. The extracted RNA was reverse transcribed into complementary DNA (cDNA) for gene cloning.

Wild-type *A. thaliana* (Col-0) and three transgenic *A. thaliana* lines (OE3, OE8, and OE10) were used as experimental materials. Seeds were first disinfected with 75% ethanol for one minute, followed by surface sterilization with 1% NaClO3 for 10 minutes. Afterward, they were washed 3–4 times with sterile water. The sterilized seeds were then placed on 1/2 MS solid medium and incubated at 4°C in the dark for three days. Subsequently, seeds were exposed to continuous light to promote germination. Once the plants reached the four-leaf stage, they were transplanted into German K brand 876 peat soil and cultivated in an artificial climate chamber at 22°C under a cycle of 16 hours light and 8 hours darkness. The plants were watered regularly and sprayed with nutrients.

### Quantitative Real-Time Polymerase Chain Reaction (qRT-PCR) And Bioinformatics Analysis

RNA was extracted from various parts of maize under unstressed conditions at the three-leaf and one-heart stages, using the TRIzol method. After the extracted RNA was reverse-transcribed into cDNA, the expression of *ZmNF-YB10* was analyzed by real-time fluorescence quantitative PCR.^38^ Once the site with the highest expression was identified, samples were collected under different stress treatments (salt, alkali, drought, and cold) during the three-leaf stage. RNA was extracted, reverse-transcribed into cDNA, and subjected to qRT-PCR using the SYBR Premix Ex Taq (Takara). The data were analyzed using the 2^−ΔΔCT^ method, with ACTIN1 serving as the internal reference gene for calculating the Ct values. Three biological replicates were used to assess the accuracy.

Genome files in FASTA format and gene annotation files in GFF3 format were obtained from Phytozome v13 for maize, sorghum, and rice. The screened *NF-YB* gene families were analyzed for covariance within maize species and between maize, sorghum, and rice species using the MCScanX tool in TBtools.Phylogenetic trees were constructed using the maximum likelihood method in IQ-TREE software, with muscle comparisons and visual enhancements made via the Evolview website.

### Generation of Constructs and Transgenic Plants

To obtain the overexpressed *ZmNF-YB10* gene, the genome sequence of maize, *ZmNF-YB10*, was downloaded from the NCBI website. Maize B73 cDNA was used as a template for cloning to isolate the *ZmNF-YB10* gene and construct the pEASY-T1-ZmNF-YB10 cloning vector. The pCAMBIA3301-ZmNF-YB10 homology arm primers were designed in combination with the pCAMBIA3301 vector to create the pCAMBIA3301-ZmNF-YB10 vector (Table S1). The CaMV35S:ZmNF-YB10 construct was transformed into *Agrobacterium* rhizogenes GV3101 and the recombinant plasmid was subsequently introduced into wild-type *A. thaliana* (Col-0) using the floral-dip method.^39^ Transgenic *ZmNF-YB10* plants were screened using glufosinate ammonium (5 mg/L, Sigma) and validated by PCR and a test strip assay, resulting in T3 generation of *Arabidopsis* plants containing the transgenic *ZmNF-YB10* gene.

### ZmNF-YB10 Protein-Induced Expression and Detection by Western Blotting

The full-length cDNA of *ZmNF-YB10* was amplified and inserted into the pET-22b vector driven by the CaMV35S promoter. The recombinant expression vector, pET-22b-ZmNF-YB10 (Table S1), was used to transform competent BL21 *Escherichia coli* cells. Protein expression was induced using isopropyl β-d-thiogalactopyranoside (IPTG) and the size of the target protein was determined by sodium dodecyl sulfate-polyacrylamide gel electrophoresis (SDS-PAGE).

Western blotting^40^ experiments were conducted for protein transfer. The PVDF membrane was immersed in methanol for one minute and then immediately rinsed with distilled water. The solution was subsequently transferred to a pre-cooled transfer buffer and equilibrated for 10 minutes. After completing the PAGE, the SDS-PAGE gel was removed and the concentrated gel was gently scraped off. A corner of the gel was marked to indicate the order of sampling. The separation gel was carefully transferred to a pre-cooled transmembrane buffer on ice for approximately 10 minutes to equilibrate ionic strength. A sandwich transmembrane structure was used, with the membrane placed in the transmembrane device in the following order from top to bottom: transmembrane template (positive), sponge, filter paper, PVDF membrane, acrylamide gel, filter paper, sponge, and transmembrane template (negative). The membrane was allowed to flow at a constant current of 200–250 mA for 2 hours. It was then placed in the containment solution, shaken on a shaker, and sealed for 1–2 hours at room temperature (or overnight at 4°C). The appropriate proportion of primary antibody was added and allowed to hybridize for 2 hours at room temperature (or overnight at 4°C), followed by washing the membrane 5 to 6 times. The secondary antibody solution was then added for hybridization for 1–2 hours at room temperature, followed by washing of the membrane 5–6 times. Chemiluminescence was measured in a dark room. Immediately after the development, the X-ray film was immersed in a fixing solution for 5–10 min until the film became transparent. The residual fixing solution was rinsed with tap water and the film was air-dried at room temperature to retain the image.

### *Tolerance Analysis of Maize ZmNF-YB10 in* E. Coli

Single colonies of newly activated BL21 (pET-22b) and control BL21 (pET-22b-ZmNF-YB10) were collected separately, inoculated into LB liquid medium, and incubated overnight at 37°C with shaking. The culture was then expanded to 50 mL, and when the optical density at 600 nm (OD_600_) of the expanded bacterial solution reached 0.4–0.6, the inducer IPTG was added to continue incubation for an additional 16 hours. At the end of the incubation period, the OD_600_ of the bacterial solution was adjusted to 0.6, and the bacteria were transferred to LB culture medium containing NaCl and mannitol at a ratio of 1:1000 for the stress experiment. The bacteria were incubated at 37°C with shaking at 180 rpm for 12 hours. Samples of the bacterial solution were taken at two-hour intervals to measure the OD_600_ value, and the growth curves were subsequently plotted.^41^

### Heterologous Expression of the Maize ZmNF-YB10 Gene in Yeast

The pYES2-ZmNF-YB10 homology arm primers (Table S1) were designed using EcoRI and HindIII as the enzyme cleavage sites. The pEASY-T1-ZmNF-YB10 plasmid served as a template for constructing *Saccharomyces cerevisiae* expression vectors. These vectors were transfected into yeast cells and cultured in SD/Ura solid medium.^42^ Single colonies were selected and incubated for 48 hours in a liquid medium. The bacterial solution was diluted in a series of 10°, 10^−1^, 10^−2^, 10^−3^, 10^−4^, 10^−5^ times, and 10 µL of yeast solution at different dilution ratios was spotted onto basal medium (SD-Ura), salt-stress medium (SD-Ura + NaCl), and drought-stress medium (SD-Ura + Mannitol) solid plates to observe growth. After screening for the optimal concentration gradient, yeast liquid was added to the liquid medium, after various stress treatments. The OD_600_ values were measured at 0, 24, 48, 72, 96, and 120 hours, and yeast growth curves were plotted.

### *Germination Rate and Phenotypic Detection Under Different Stress Treatments in* A. thaliana

In seed germination assays involving various stress treatments (salt and drought), both overexpressed and wild-type (Col-0) seeds were first washed with 75% ethanol for one minute, sterilized with 1% hypochlorite for 10 minutes, and subsequently rinsed 3–4 times with sterile water. The seeds were then placed on salt (300 mm NaCl), mannitol (300 mm), and untreated 1/2 MS solid media, and incubated for three days at 4°C in the dark, followed by two weeks at 22°C under continuous light to assess germination percentages.

The T3 generation of *A. thaliana* was planted in nutrient-rich soil, and growth was monitored and recorded at four weeks of age. Wild-type *Arabidopsis* (Col-0) and overexpression lines were evaluated for various phenotypes, including rosette leaf area, plant height at seven weeks, fruit pod length, and number of flower buds. The rosette leaf area was calculated by averaging the longest and shortest leaf spans of a pair of leaves using the formula for the area of a circle. Phenotypic changes in wild-type *Arabidopsis* (Col-0) and overexpression plants (OE3, OE8, and OE10) were photographed and documented at four weeks of age under drought and salt stress conditions.

### Activated Oxygen and Biochemical Indicator Tests

Nitroblue tetrazolium chloride (NBT) staining solution was prepared by dissolving NBT (0.5 g) in 500 µL of sodium azide solution and 500 µL of 1 M sodium phosphate buffer, resulting in a total volume of 50 mL. Additionally, diaminobenzidine (DAB) staining solution was prepared by mixing 20 mg DAB with 38 mL double-distilled water, and the pH was adjusted with 0.2 M HCl to achieve a final concentration of 0.5 mg/mL. Four-week-old *Arabidopsis* leaves subjected to various stress treatments (salt and drought), as well as untreated *Arabidopsis* leaves, were immersed in NBT/DAB staining solution and incubated for eight hours. After discarding the staining solution, samples were submerged in 95% ethanol and placed in a water bath at 95°C for decolorization. Once the green color of the samples had completely faded, photographs were taken to document the staining results.

Assay kits for H_2_O_2_,^43^ superoxide anion (O^2-^),^44^ catalase (CAT),^45^ peroxidase (POD),^46^ SOD,^43^ and MDA^43^ were used to measure the activity of the samples.

### Yeast Two-Hybrid Assay

The two reciprocal proteins with the highest STRING prediction scores from online databases were selected to verify the reciprocal relationship between ZmNF-YC2, ZmNF-YC4, and ZmNF-YB10 through yeast two-hybrid experiments. First, a yeast toxicity test was performed. The constructed bait vector pGBKT7-ZmNF-YB10 plasmid and empty bait vector pGBKT7 were introduced into AH109 yeast receptor cells, which were then plated on a selective medium deficient in tryptophan (SD/-Trp) and incubated at 29°C for 2–3 days to observe the growth and distribution of the proteins, thereby determining whether the proteins exhibited toxicity. Subsequently, self-activation was assessed. The constructed bait vector pGBKT7-ZmNF-YB10 was co-transfected with the empty prey vector pGADT7 into AH109 yeast receptor cells, which were then plated on a medium deficient in SD/-Trp-Leu and incubated at 29°C for 2–3 days. A single colony was selected for further culture in the YPDA medium. The positive control, negative control, and experimental groups were spotted on media deficient in SD/-Trp-Leu, as well as on four deficient media (SD/-Trp-Leu-Ade-His) and (SD/-Trp-Leu-Ade-His) supplemented with X-α-Gal. The growth of the experimental group was monitored to determine the self-activating activity of the ZmNF-YB10 protein.

The constructed bait vector pGBKT7-ZmNF-YB10 and prey vectors pGADT7-ZmNF-YC2 and pGADT7-ZmNF-YC4 were co-transfected into AH109 yeast sensory cells, which were cultured in synthetic dropout medium lacking SD/-Trp-Leu at 29°C for 2–3 days. Single colonies were selected and cultured in a YPDA medium. When the optical density at 600 nm (OD_600_) reached 0.6, 10 μL of the bacterial solution was added to the positive control, negative control, and experimental groups on two-deficient (SD/-Trp-Leu), four-deficient (SD/-Trp-Leu-Ade-His), and four-deficient (SD/-Trp-Leu-Ade-His) + X-α-Gal media for further analysis. The experimental group was incubated for an additional 3–4 days at 29°C to observe growth conditions. After this period, the growth of the experimental groups was assessed to determine whether a mutualistic relationship existed between ZmNF-YB10, ZmNF-YC2, and ZmNF-YC4.

### Data Analysis

All experiments were performed in triplicate and the data are presented as the mean ± standard error of the mean from three biological replicates. Data were analyzed for significance using a one-way analysis of variance with the biostatistical software SPSS, version 25.0. Different letters indicate significant differences at 0.05, whereas * indicates significant differences at 0.05, and ** indicates highly significant differences at a 0.01 level (* *p* < .05, ** *p* < .01).

## Results

### Bioinformatics Analysis of the ZmNF-YB10 Gene

**Figure 1.**
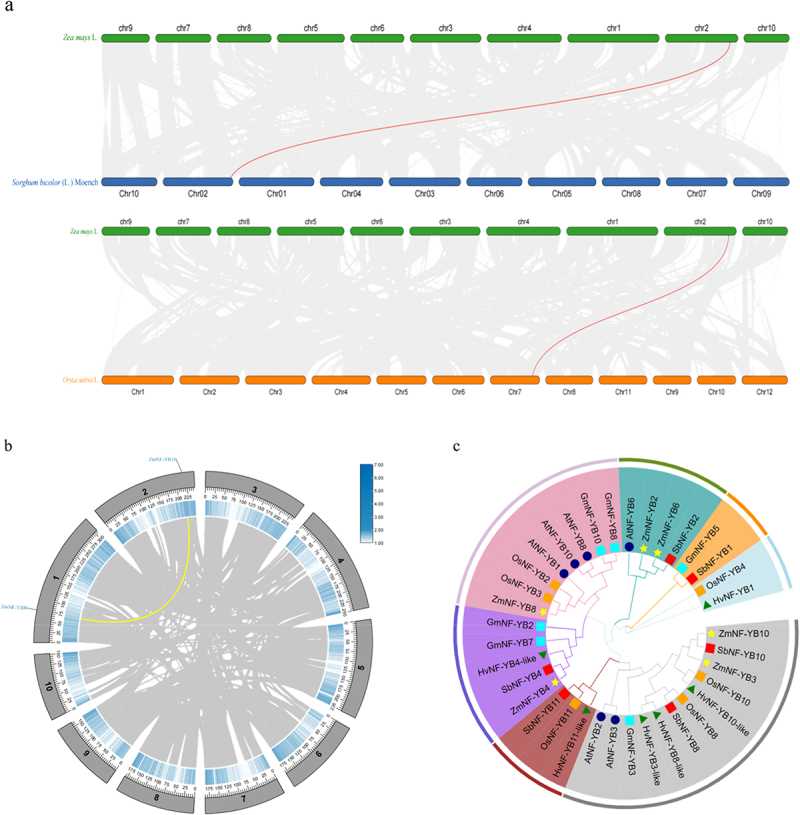
Bioinformatics analysis of *ZmNF-YB10* gene. (a) *ZmNF-YB10* interspecies covariance analysis. (b) *ZmNF-YB10* intraspecies covariance analysis. (c) *ZmNF-YB10* phylogenetic tree analysis.

In this study, the maize *ZmNF-YB10* gene was successfully cloned from maize inbred line B73, which served as the recipient material. *ZmNF-YB10* has a total length of 1209 base pairs (bp) and a coding sequence (CDS) region of 618 bp, encoding a polypeptide composed of 205 amino acid residues with a predicted molecular weight of 21.81 kDa and an isoelectric point value of 5.85 (Table 1). Protein structure analysis revealed that the gene belongs to the NF-Y and NF-YB subfamilies; one structural domain was predicted based on the conserved structural domains. There was no transmembrane domain or signal peptide and the encoded protein was hydrophilic. Interspecies covariance analysis indicated covariance between *ZmNF-YB10*, *OsNF-YB10*, and *SbNF-YB3* (Figure 1a). Intraspecific covariance analysis showed covariance between *ZmNF-YB10* and *ZmNF-YB8* (Figure 1b). The results of evolutionary tree analysis indicated that *ZmNF-YB10* was most closely related to *SbNF-YB10* and *ZmNF-YB3* (Figure 1c).

**Table 1.** The physiochemical characteristics of maize ZmNF-YB10 proteins.

Gene name	Gene ID	CDS length（bp）	Protein length（aa）	MW（KDa）	PI	Chr
ZmNF-YB10	Zm00001d006813	618	205	21.81	5.85	2

### Expression Pattern Analysis of the ZmNF-YB10 Gene

**Figure 2.**
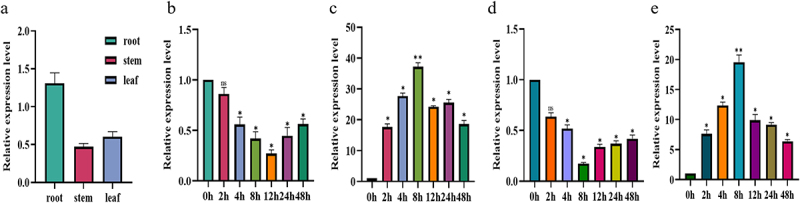
Analysis of *ZmNF-YB10* gene expression pattern. (a) Differential expression analysis of *ZmNF-YB10* in different maize tissues. (b) Expression pattern analysis of *ZmNF-YB10* in roots under alkali treatment (125 mm/L NaHCO3: Na2CO3 = 9:1). (c) Expression pattern analysis of *ZmNF-YB10* in roots under drought (300 mm/L mannitol). (d) Expression pattern analysis of *ZmNF-YB10* in roots under cold (4°C) treatment. (e) Expression pattern analysis of *ZmNF-YB10* in the roots under salt (300 mm/L NaCl) treatment. Student’s *t*-test was performed; asterisks indicate significant differences, with *p* < .05 denoted by *and *p* < .01 denoted by **. Data are expressed as the mean±standard deviation of three independent tests.

Expression analysis of various parts of maize at the three-leaf stage revealed that *ZmNF-YB10* exhibited the highest expression levels in the roots (Figure 2a). Subsequently, different stresses (cold, salt, alkali, and drought) were applied to the maize roots at the three-leaf stage. The results indicated that plants responded most significantly to salt and drought stress treatments, demonstrating a positive feedback expression pattern under these conditions (Figure 2b–e).

### Expression of Maize ZmNF-YB10 in Prokaryotic Systems

**Figure 3.**
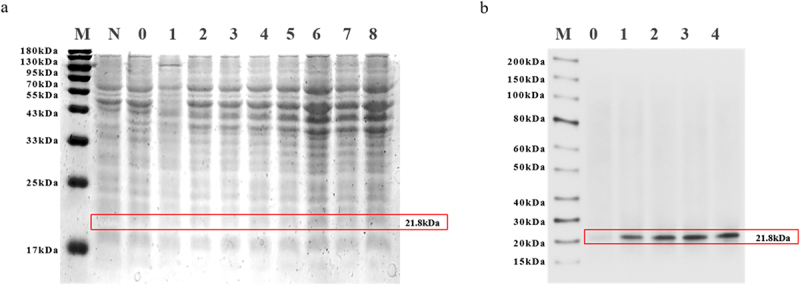
Efficient expression of *ZmNF-YB10* in prokaryotic systems. (a) SDS-page electrophoretic analysis of the *ZmNF-YB10* recombinant protein. M, protein marker; N, pEt22b empty vector not induced by IPTG; 0, pEt22b-ZmTCP14 non-induced bacteria; 1–8, pEt22b-ZmNF-YB10 induced for 1–8 h. (b) Protein blot of ZmNF-YB10. M: protein marker; 0: pEt22b-ZmNF-YB10 induced bacteria; 1–4: pEt22b-ZmNF-YB10 induced bacteria.

We first constructed the prokaryotic expression vector pET-22b-ZmNF-YB10 and transferred it into *E. coli* BL21. Protein expression was induced by adding 0.1 mm IPTG when the OD_600_ of the bacterial culture reached approximately 0.4–0.6. The samples were placed in a shaker at 28°C and collected every 2 hours. SDS-PAGE results indicated that the protein band was successfully observed within the molecular weight range of 25–33 kDa (Figure 3a), which was consistent with the predicted size of ZmNF-YB10, confirming its successful expression in BL21. Before SDS-PAGE, the gel was transferred to a membrane and incubated with primary and secondary antibodies, resulting in band formation using DAB color development. Western blot analysis of the pET-22b-ZmNF-YB10 vector revealed a distinct band at 17–25 kDa (Figure 3b). Therefore, we successfully induced the expression in maize *ZmNF-YB10*.

### *Tolerance Analysis of Maize ZmNF-YB10 in* E. Coli

**Figure 4.**
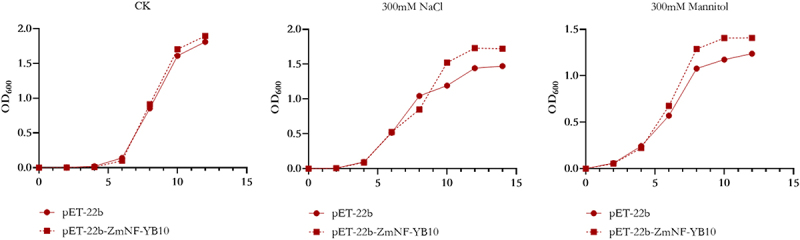
Salt and drought tolerance analyses of *ZmNF-YB10* in *E. coli* BL21.

Recombinant and empty pET-22b plasmids were transferred into *E. coli* BL21, induced with 0.2 M IPTG and subjected to simulated stress experiments using 300 mm NaCl and mannitol. The growth curves are shown in Figure 4. Under normal conditions, no significant growth was observed in BL21 (pET-22b) or BL21 (pET-22b-ZmNF-YB10) cells. However, under drought and salt stress conditions, the growth of both bacterial strains was inhibited. Notably, the growth inhibition rate of BL21 (pET-22b) was significantly higher than that of BL21 (pET-22b-ZmNF-YB10) (Figure 4). These results suggest that the expression of *ZmNF-YB10* enhances drought and salt tolerance in *E. coli*.

### Heterologous Expression of the Maize ZmNF-YB10 Gene in Yeast

**Figure 5.**
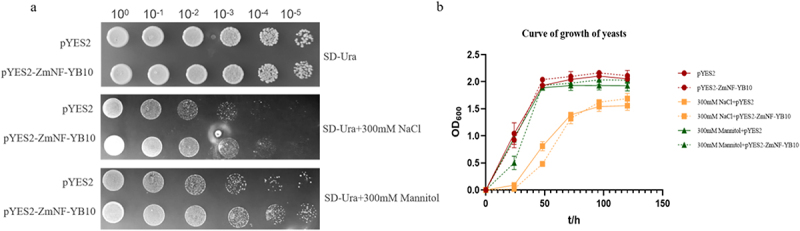
Heterologous expression of *ZmNF-YB10* in yeast cells. (a) Tolerance analysis of *ZmNF-YB10* in yeast under abiotic stress conditions. (b) Yeast growth curves.

In this study, a pYES2-ZmNF-YB10 expression vector was constructed, and both the expression vector and empty vector were transferred into INVSc1 yeast strains. These strains were cultured in basal, salt stress, and drought stress media to assess their growth. The results demonstrated that the growth of yeast strains containing the pYES2-ZmNF-YB10 vector was significantly better than that of strains containing the empty pYES2 vector under both salt and drought stress conditions (Figure 5a). This suggests that maize *ZmNF-YB10* enhances salt and drought tolerance in the yeast.

Yeast cells containing empty carriers and those transformed with *ZmNF-YB10* were cultured in basal (SD-Ura), salt stress, and drought stress media. The absorbance values at OD_600_ were measured at 0, 24, 48, 72, 96, and 120 hours, and growth curves were plotted. The results indicated that the growth potentials of yeast with the pYES2-empty vector and those transformed with the pYES2-ZmNF-YB10 vector were comparable in the basal medium. However, under salt and drought stress conditions, the growth potential of yeast transformed with the pYES2-ZmNF-YB10 vector was superior to that of yeast transformed with the pYES2-empty vector (Figure 5b). This finding suggests that maize *ZmNF-YB10* exhibits a biological function that enhances salt and drought tolerance in yeast cells.

### *Germination and Phenotypic Analysis of ZmNF-YB10 Gene in* A. thaliana *Under Different Stress Conditions*

To gain a deeper understanding of the role of *ZmNF-YB10* under drought and salt stress conditions, we conducted a genetic transformation of *A. thaliana*. We successfully obtained three transgenic plants designated OE3, OE8, and OE10, which exhibited high expression levels of 35S:NF-YB10. These plants were subsequently analyzed for germination rate and phenotypic characteristics under various stress treatments.

**Figure 6.**
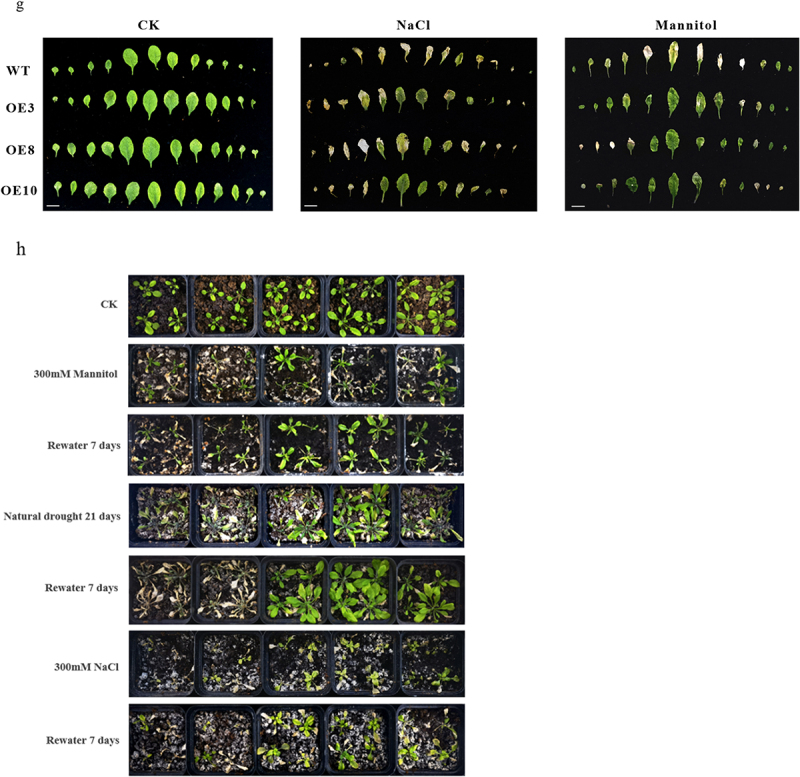
Continued

First, we observed that both wild-type and transgenic *A. thaliana* grew normally, with no significant difference in seed germination rate in the control 1/2 MS medium. However, in 1/2 MS medium containing 300 mmol/L NaCl and 300 mmol/L mannitol, the seed germination rate of the transgenic plants was superior to that of the wild-type (Figure 6a).
**Figure 6.**
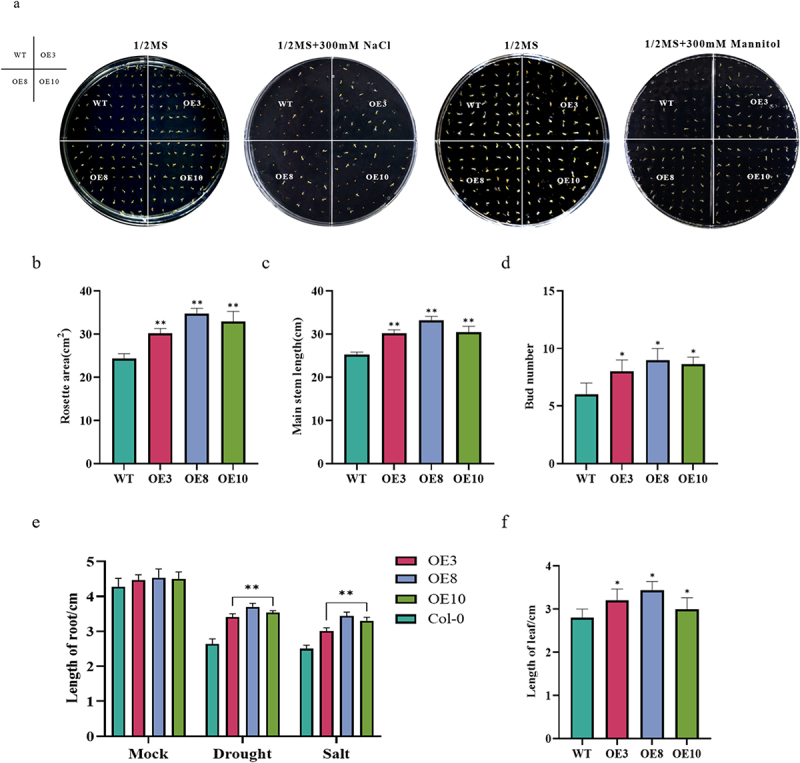
*Arabidopsis* germination rate and phenotypic determinations: (a) germination rate, (b) rosette leaf area, (c) plant height, (d) fruit pod size, (e) root length, (f) length of the sixth leaf blade, (j) leaf changes under stress treatments, (k) leaf length, and (g) leaf phenotypic changes under drought and salt stress treatments. (h) Phenotypic changes in transgenic *Arabidopsis* plants under drought and salt stress treatments. The Student’s *t*-test was performed; asterisks indicate significant differences, with *p* < .05 denoted by * and *p* < .01 denoted by **. Data are expressed as the mean ± SD of three independent experiments.

Next, we examined rosette leaf area, plant height, fruit pod size, number of flower buds, root length, and leaf length in four-week-old wild-type *Arabidopsis* and *Arabidopsis* overexpressing *ZmNF-YB10*. The results indicated that rosette leaf area (Figure 6b), plant height (Figure 6c, Figure S1), fruit pod size (Figure S2), number of bracts (Figure 6d, Figure S3), root length (Figure 6e, Figure S4), and leaf length (Figure 6f) were significantly greater in the overexpression lines than in the wild-type. After three consecutive weeks of drought (300 mm/L mannitol and natural drought) and high-salt (300 mmol/L NaCl) stress treatments on both wild-type and transgenic plants, we observed that the leaves of wild-type *Arabidopsis* were severely wilted and dehydrated, whereas the leaves of the overexpressing plants were only partially wilted and exhibited healthier growth (Figure 6g). Seven days after re-watering, only 25% survival was recorded in stress-treated wild-type *Arabidopsis*, whereas approximately 80% survival was observed in transgenic plants under drought stress and approximately 75% survival was observed in transgenic plants subjected to salt stress treatment (Figure 6h, Figure S5). Therefore, we hypothesized that this gene is positively regulated in maize in response to drought and salt stress.

### Overexpression of ZmNF-YB10 Affects ROS Homeostasis

**Figure 7.**
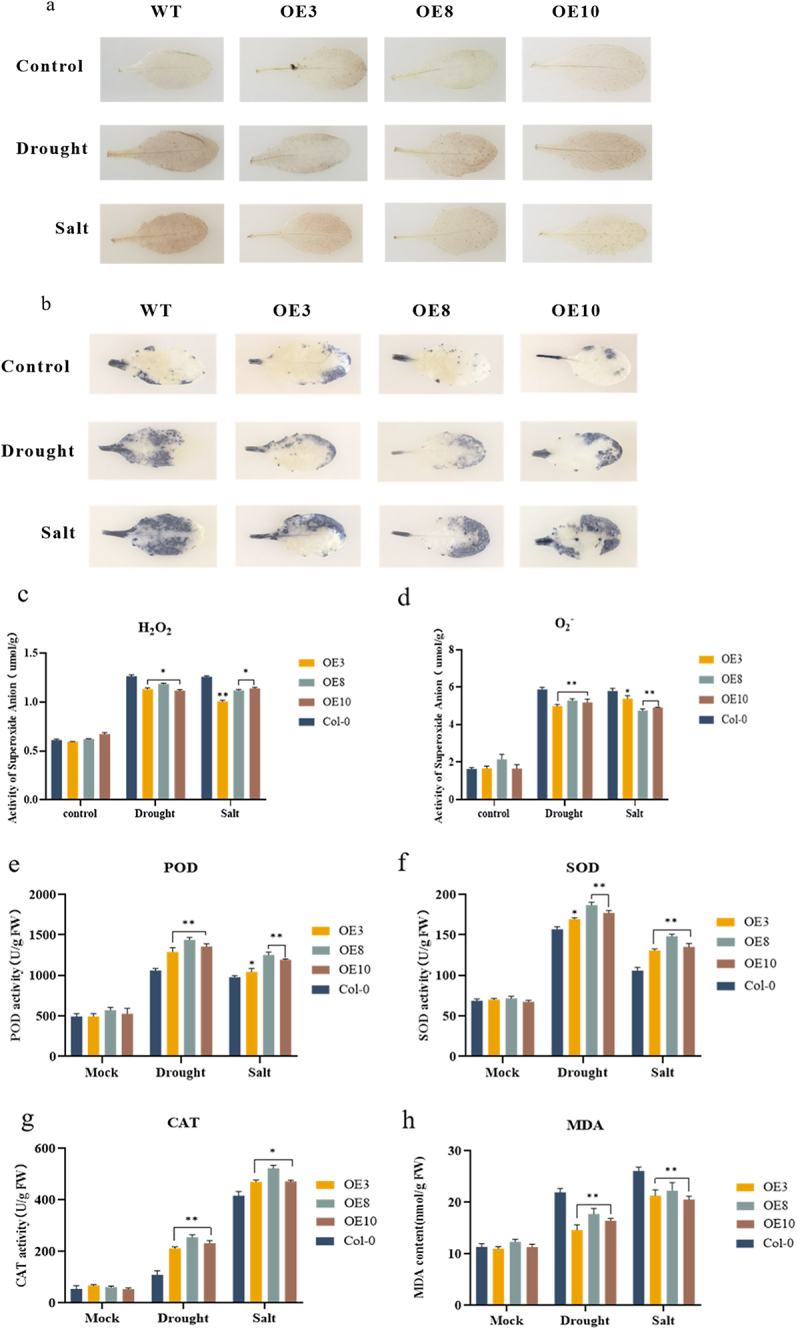
ROS staining and physiological index detection in *ZmNF-YB10 Arabidopsis* plants. (a) DAB staining. (b) NBT staining. (c-d) accumulation of H_2_O_2_ and O^2 -^ in the leaves of different strains. (e-h) POD, SOD, CAT activities, and MDA assays were determined in *Arabidopsis* under different stress treatments. The Student’s *t*-test was performed; asterisks indicate significant differences, with *p* < .05 denoted by * and *p* < .01 denoted by **. Data are expressed as mean ± SD of three independent tests.

DAB and NBT staining of *Arabidopsis* leaves revealed that under normal conditions, the staining effects of wild-type and overexpressing plants were comparable, with no significant differences observed. However, the leaves of overexpressing plants exhibited lighter staining than those of wild-type plants under drought and salt stress, as indicated by reduced dark blue and yellow-brown precipitation. This suggested that the levels of H_2_O_2_ and O_2_ in the overexpression lines were reduced under stress conditions (Figure 7a,b). The H_2_O_2_ and O_2_ contents were measured and the results were consistent with the staining observations (Figure 7c,d). Additionally, the activities of SOD, POD, and CAT, as well as the MDA content, were assessed in both unstressed and stress-treated (salt and drought) wild-type and transgenic *A. thaliana*. The findings revealed that the SOD, POD, and CAT activities in *A. thaliana* transgenic for *ZmNF-YB10* were significantly higher than those in the wild-type, whereas the MDA content was significantly lower under both stress treatments. This indicates that the expression of *ZmNF-YB10* enhances the survival capacity of *Arabidopsis* under drought and salt stress conditions (Figure 7e–h).

### *Expression Analysis of Resistance-Related Genes in Overexpressing* A. thaliana

Because transgenic *ZmNF-YB10* Arabidopsis enhances drought and salt tolerance, we used qRT-PCR to analyze the drought-associated marker gene DRE-binding protein 2A (*AtDREB2A*, AT5G05410), senescence-associated gene 13 (*AtSAG13*, AT2G29350), cold-regulated gene 15 (*AtCOR15*, AT2G42540), and salt tolerance-associated gene SAL1 phosphorothionein (*AtCOR15*, AT2G42540) in drought and salt stress-treated transgenic plants. associated gene 13 (*AtSAG13*, AT2G29350), cold-regulated 15 (*AtCOR15*, AT2G42540), salt tolerance-associated gene phosphatase-like protein FRY1(*AtFRY1*, AT5G63980), small nuclear ribonucleoprotein family protein (*AtSAD1*, AT5G48870), and protein kinase superfamily protein (*AtSOS2*, AT5G35410). It was found that the expression of *AtSAG13*, *AtCOR15*, *AtDREB2A*, *AtFRY1*, *AtSAD1*, and *AtSOS2* in Arabidopsis and wild-type plants was significantly higher than that in wild-type plants after stress treatment. (Figure S6). These results suggest that the overexpression of *ZmNF-YB10* in *Arabidopsis* enhances the expression of relevant marker genes.

### Toxicity Validation and Self-Activation Assay of the Decoy Vector (pGbkt7- ZmNF-YB10) in Yeast Cells

**Figure 8.**
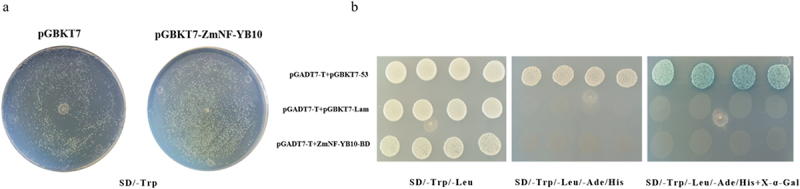
Toxicity validation and self-activation assay of *ZmNF-YB10* in yeast cells.

The results of the yeast toxicity assay indicated that the growth status, growth rate, and number of yeast cells transformed with the bait protein on one-deficient selection medium were consistent with those of the control, showing no significant differences. This suggests that the bait vector was not toxic (Figure 8a). In addition, the results of the self-activation assay revealed that white plaques formed on the two-deficient selection medium but did not turn blue on the four-deficient selection medium coated with X-α-Gal. This indicated that the bait vector exhibited no self-activation activity (Figure 8b), and subsequent experiments were performed.

### Validation of the Interaction Between ZmNF-YB10, ZmNF-YC2, and ZmNF-YC4 Proteins

**Figure 9.**
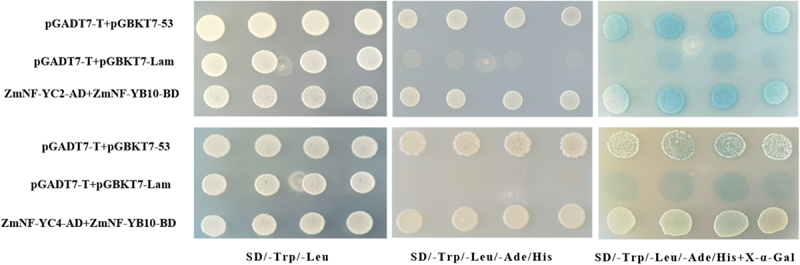
Validation of *ZmNF-YB10* interaction in yeast.

After co-transforming pGADT7-ZmNF-YC2 and pGBKT7-ZmNF-YB10, as well as pGADT7-ZmNF-YC4 and pGBKT7-ZmNF-YB10, into tetra-deficient plates containing X-α-Gal, the yeast strains exhibited robust growth and turned blue. This observation suggests that *ZmNF-YB10* interacts with the proteins expressed by *ZmNF-YC2* and *ZmNF-YC4* in yeast (Figure 9). It is hypothesized that *ZmNF-YC2* and *ZmNF-YC4* play significant roles in the response of maize to stress conditions.

## Discussion

Drought and salt stress are environmental challenges that deplete soil water reserves in many regions worldwide. The salinization of arable land is increasing dramatically owing to the ongoing climate change.^42^ Multiple factors, including environmental changes, influence various traits, such as crop yield and quality; thus, the mechanisms by which plants respond to abiotic stress are highly complex. Previous studies have demonstrated that transcription factors (TFs) are key regulators of stress responses and excellent candidates for crop improvement. These transcription factors play a central role in gene regulatory networks that mediate various aspects of plant developmental processes and their responses to environmental changes.^47^ TFs regulate plant functions related to environmental factors and hormones as well as aspects of cell differentiation and organ development.

In this study, we identified and characterized a transcription factor gene, *ZmNF-YB10*, that is associated with stress tolerance. One of the most significant advancements in plant drought response over the past decade has been the identification of ABA^48^ receptors and the elucidation of the ABA signaling pathway.^49^ Recent studies have demonstrated that NF-Ys play a key role in the regulation of the ABA signaling pathway. The *PdNF-YB21* transcription factor interacts with *PdFUS3* and activates the key gene for ABA synthesis, *PdNCED3*, leading to an increase in ABA content, enhanced transport of growth hormones in the roots, and ultimately, improved drought tolerance in poplar. Under drought stress conditions, overexpression of *ZmNF-YB16*^32^ significantly increases the resistance and yield of maize plants during both nutritional and reproductive stages. Additionally, *AtNF-YA2*, *AtNF-YA3*, *AtNF-YA5*, *AtNF-YA7*, and *AtNF-YA10*^50^ have been shown to enhance drought tolerance in *A. thaliana*. Furthermore, NF-Y not only regulates the ABA-dependent pathway but also the ABA-independent pathway to improve drought tolerance in plants.^51^ Overexpression of the *StNF-YC9*^28^ gene in potato increases root length and photosynthetic rates while reducing water loss under short-term drought stress. The expression of the key regulatory genes *TaCAT1* and *TaPOD4* have been shown to be positively correlated with the expression of *TaNF-YA7-5B* under drought conditions. These two genes are involved in proline accumulation and ROS scavenging, with *TaNF-YA7-5B* serving as a crucial ABA-independent regulator of plant adaptation to drought.^19^ Due to the complexity of plant traits and the numerous factors influencing them under drought conditions, NF-Y can exhibit either enhanced or reduced drought tolerance based on stress-related parameters, including chlorophyll content,^52^ stomatal conductance, and leaf temperature. In contrast, salt stress is a significant factor leading to stunted growth, reduced height, and impaired reproduction in plants. Recent studies have reported the mechanisms by which NF-Y responds to salt stress; for instance, *NF-YC9* promotes the expression of *SRMT*-regulated genes and enhances salt tolerance in poplar.^53^ Overexpression of *SiNF-YA1*^54^ in transgenic tobacco lines enhances drought and salt tolerance. *Arabidopsis* plants that heterologously express wild spruce *PwNF-YB3*^27^ exhibit significantly greater tolerance to salt, drought, and osmotic stress. Because drought and osmotic stress are often accompanied by salt stress,^55^ it is essential to consider the response to both stresses when searching for genes involved in salt tolerance.^56^ In this study, the results of stress tolerance analysis in transgenic *A. thaliana* indicated that *ZmNF-YB10* transgenic plants showed enhanced tolerance to mannitol and NaCl stress treatments. We also examined the proline content, ROS levels, and antioxidant enzyme activities. POD plays multiple roles in the antioxidant and redox signaling networks of cells, whereas CAT is a key enzyme in the biological defense system that provides organisms with an antioxidant defense mechanism. SOD is an enzyme that scavenges superoxide anion radicals. An adverse environment can lead to the accumulation of superoxide anion radicals, which in turn induce the production of SOD in plants. MDA content serves as an indicator of the degree of peroxidation in the cytoplasm of plant cells; elevated MDA levels signify a higher degree of peroxidation, resulting in significant damage to the cell membranes. In this study, under 300 mm NaCl and 300 mm mannitol stress, POD, CAT, and SOD activities in 35S:ZmNF-YB10 *A. thaliana* were significantly higher than those in the wild-type plants. Additionally, the MDA content was lower in the transgenic plants than in the wild-type plants, whereas the levels of H_2_O_2_ and O^2^-) exhibited a decreasing trend relative to the wild-type. These results suggest that *ZmNF-YB1*0 positively influences plant responses to salinity, osmotic, and drought stress.

In addition, because most of our studies on the function of NF-Ys are still at the histological level, understanding the mechanisms and functions of NF-Y complexes is crucial for future research. Proteins that interact with complex networks perform various targeting functions. The maize NF-Y family gene *NF-YA3*^31^ can interact with the jasmonic acid activator *MYC4* to enhance drought and heat tolerance. *ZmNF-YA1* and *ZmNF-YB16*^35^ synergistically regulate maize growth and drought resistance. Therefore, we conducted a yeast two-hybrid assay to verify the interactions of *ZmNF-YB10* with *ZmNF-YC2* and *ZmNF-YC4*. It was hypothesized that *ZmNF-YB10* may also perform diverse targeting functions by interacting with *ZmNF-YC2* and *ZmNF-YC4*. This information is highly relevant for the application of NF-Ys in agricultural practices, particularly molecular breeding. These findings contribute to a better understanding of the role of NF-Y transcription factors in abiotic stress response.

## Conclusions

The maize NF-Y family gene, *ZmNF-YB10*, has a full cDNA length of 618 bp, encodes 205 amino acids, and has a molecular weight of 21.8 kDa. This gene was expressed in various tissues and organs of maize, with the highest expression observed in the roots, where it was induced by drought, cold, salt, and alkaline stress. Under drought and salt stress conditions, the levels of H_2_O_2_, O^2^-, and MDA in the soil of transgenic *ZmNF-YB10* plants were reduced, whereas the activities of POD, CAT, and SOD were elevated. Protein interactions involving *ZmNF-YB10*, *ZmNF-YC2*, and *ZmNF-YC4* gene expression were identified. It is hypothesized that *ZmNF-YB10*, *ZmNF-YC2*, and *ZmNF-YC4* may play significant roles in the response of maize to drought and high salt stress.

## Supplementary Material

Supplementary Figures.docx

Supplementary Table.docx
