# Supplementary material for: ZmNF-YB10, a maize NF-Y transcription factor, positively regulates drought and salt stress response in Arabidopsis thaliana
Source: GM Crops Food. 2024 Dec 24;16(1):28–45. doi: 10.1080/21645698.2024.2438421 (PMC11702966; doi:10.1080/21645698.2024.2438421)
Supplement: Supplementary Figures.docx [file KGMC_A_2438421_SM6100.docx]

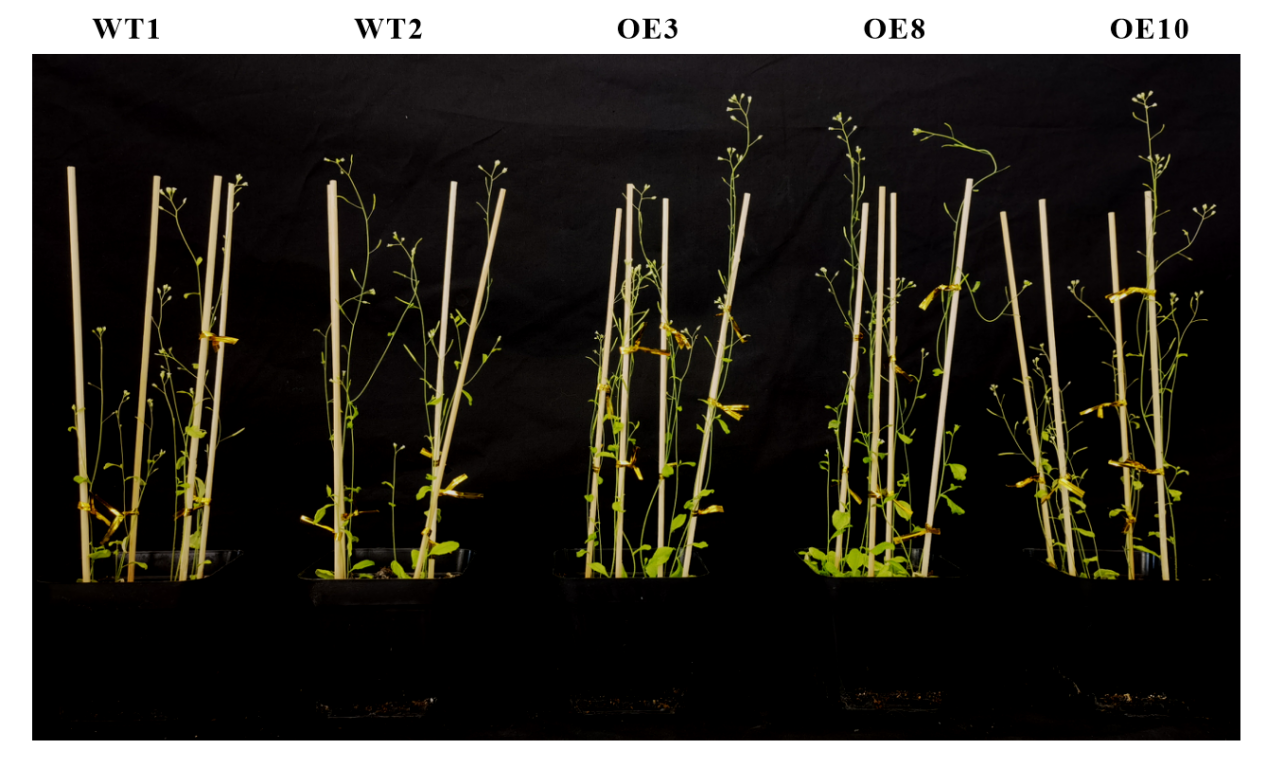


**Fig S1.** Phenotypic representation of the height of *Arabidopsis* transgenic plants for the *ZmNF-YB10* gene.


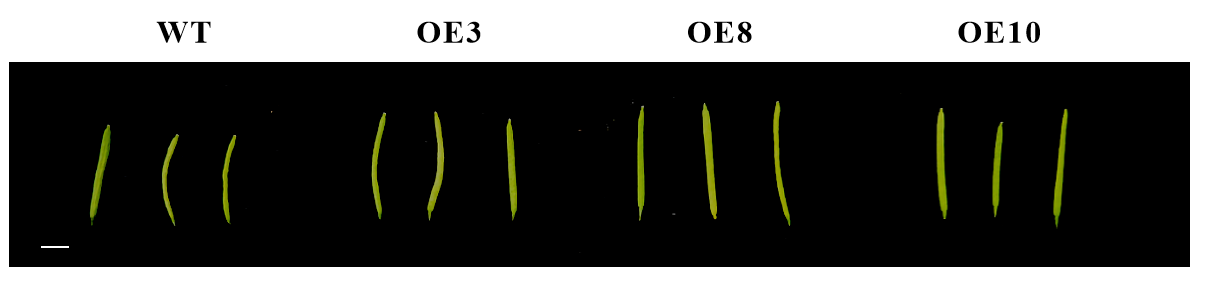


**Fig S2.** Phenotypic representation of fruit pod size in *A. thaliana* with the transgenic *ZmNF-YB10* gene.


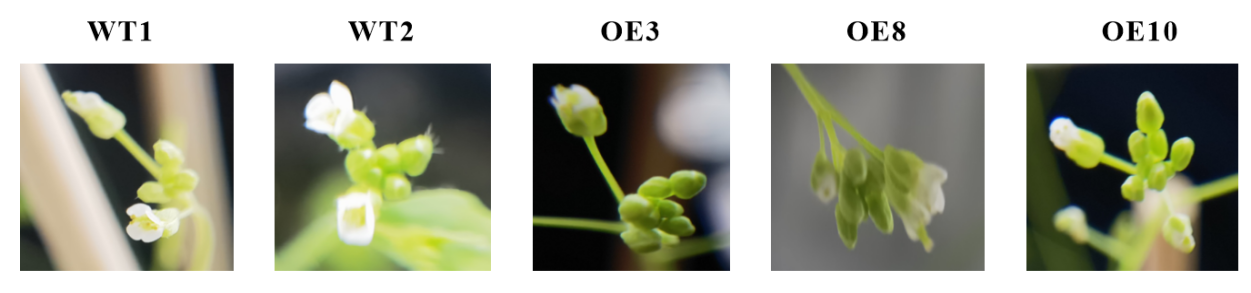


**Fig S3.**Phenotypic detection of pod numbers in A. thaliana  using Transgenic ZmNF-YB10.


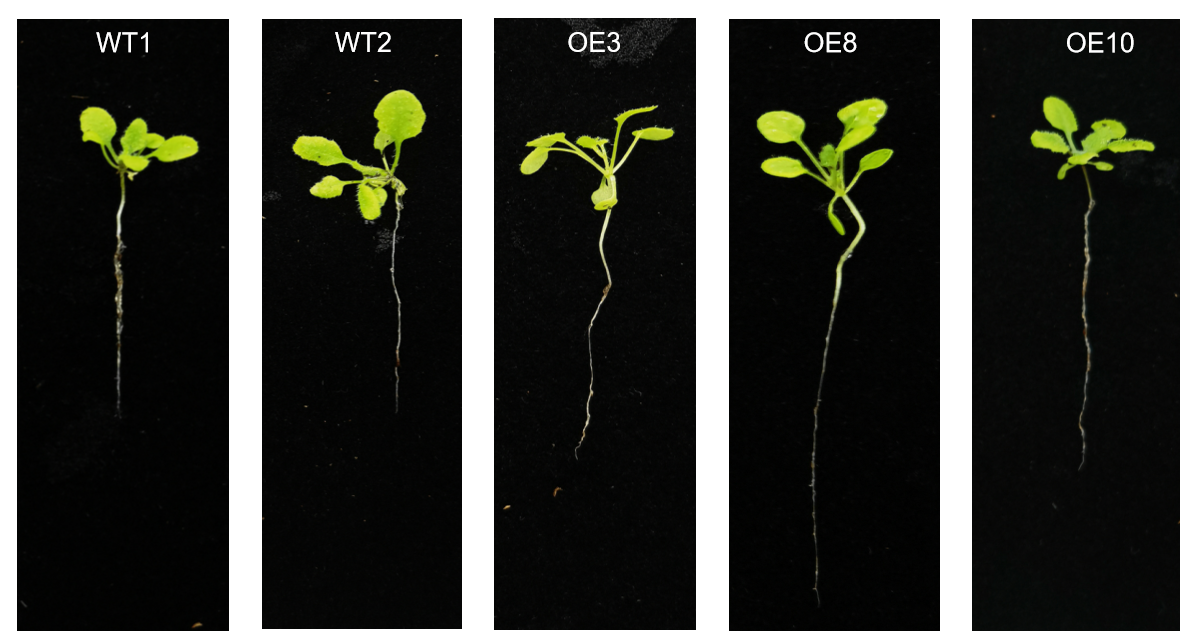


**Fig S4.** Graphical representation of the root length phenotype of *A. thaliana* with transgenic *ZmNF-YB10*.


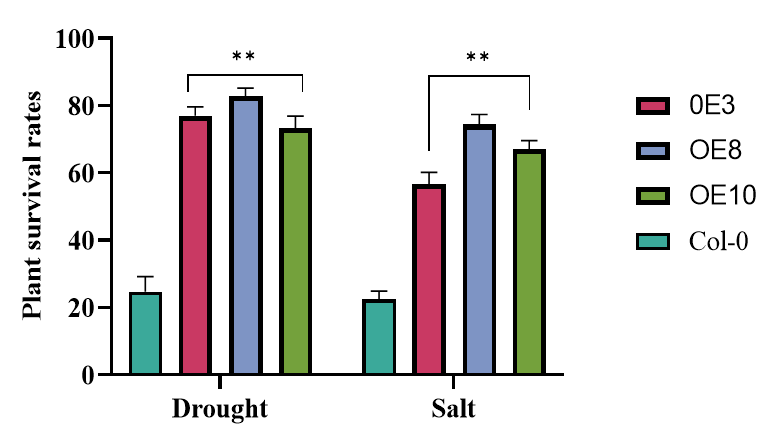


**Fig S5.** Survival of transgenic plants under drought and salt stress conditions.


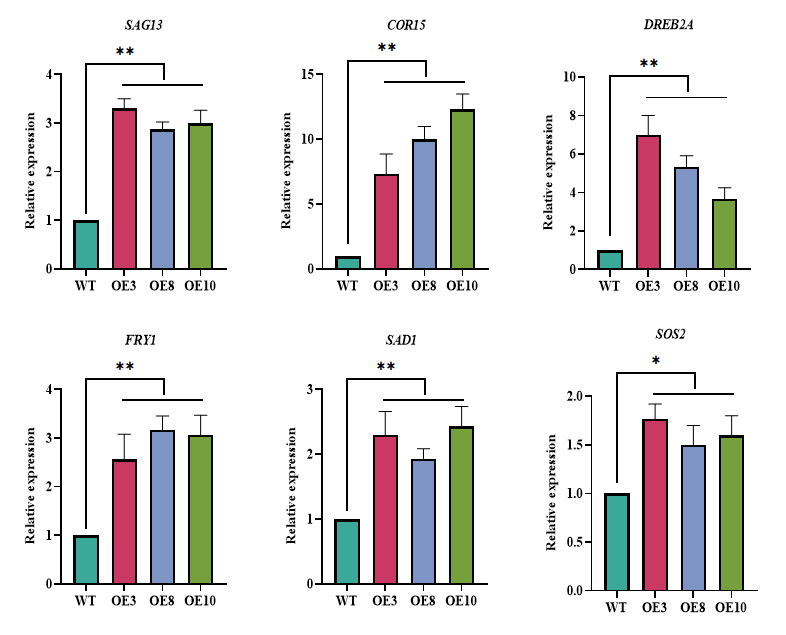


**Fig S6.** Expression of resistance-related genes in transgenic Arabidopsis thaliana
