# Supplementary material for: ZmNF-YB10, a maize NF-Y transcription factor, positively regulates drought and salt stress response in Arabidopsis thaliana
Source: GM Crops Food. 2024 Dec 24;16(1):28–45. doi: 10.1080/21645698.2024.2438421 (PMC11702966; doi:10.1080/21645698.2024.2438421)
Supplement: Supplementary Table.docx [file KGMC_A_2438421_SM6099.docx]

Table S1. Primer information in this study

| Primer name | Primer sequences（5’—3’） |
| --- | --- |
| ZmNF-YB10-F | ATGCCGGACTCCGACAACGA |
| ZmNF-YB10-R | TCACAACCCGTCGTGCCTGC |
| ZmActin1-qPCR-F | ATGTTTCCTCCCATTGCCGAT |
| ZmActin1-qPCR-R | CCAGTTTCGTCATACTCTCCCTTG |
| YG-ZmNFYB10-F | CGTGAGCCGGATCATGAAG |
| YG-ZmNFYB10-R | CTTGCCGTCCTTGGATATCTTG |
| pCAMBIA3301-ZmNF-YB10-F | actcttgaccatggtagatctATGCCGGACTCCGACAACGA |
| pCAMBIA3301-ZmNF-YB10-R | ggggaaattcgagctggtcaccTCACAACCCGTCGTGCCTGC |
| pET-22b-ZmNF-YB10-F | tcgagctccgtcgacaagcttATGCCGGACTCCGACAACGA |
| pET-22b-ZmNF-YB10-R | gtggtggtggtggtgctcgagTCACAACCCGTCGTGCCTGC |
| pGADT7-ZmNF-YB10-F | gccatggaggccagtgaattcATGCCGGACTCCGACAACGA |
| pGADT7-ZmNF-YB10-R | cagctcgagctcgatggatccTCACAACCCGTCGTGCCTGC |
| pGBKT7-ZmNF-YB10-F | atggccatggaggccgaattcATGCCGGACTCCGACAACGA |
| pGBKT7-ZmNF-YB10-R | ccgctgcaggtcgacggatccTCACAACCCGTCGTGCCTGC |
| pYES2-ZmNF-YB10-F | actatagggaatattaagcttATGCCGGACTCCGACAACG |
| pYES2-ZmNF-YB10-R | tgatggatatctgcagaattcTCACAACCCGTCGTGCCTG |
| AD-ZmNF-YC4(B4FWZ1)-F | gccatggaggccagtgaattcATGGAACCATCCTCACAGCC |
| AD-ZmNF-YC4(B4FWZ1)-R | cagctcgagctcgatggatccTCAGGCAGACTGCTGCTGCT |
| AD-ZmNF-YC2(B4FLF9)-F | gccatggaggccagtgaattcATGGAACCATCCCCTCAGCC |
| AD-ZmNF-YC2(B4FLF9)-R | cagctcgagctcgatggatccTCATGCAGATTGCTGCTCTT |
| SAG13-F | ATGGGCGAGCGACAACATAA |
| SAG13-R | GAAATGCCACAAGCGGTGAG |
| COR15-F | GGCCACAAAGAAAGCTTCAG |
| COR15-R | CTTGTTTGCGGCTTCTTTTC |
| DREB2A-F | TTGATCCGCTACAAAGCCTCA |
| DREB2A-R | CAGTCGTTGTGGGATTAAGGC |
| Actin1-F | TCGCCATCCAAGCTGTTCTC |
| Actin1-R | TCACGTCCAGCAAGGTCAAG |
| FRY1-F | CGCAGTAGCACTAGGATTG |
| FRY1-R | TTGACACCGAGTTTATTGG |
| SAD1-F | GCGAACAATCCTTCACAG |
| SAD1-R | CTTCGGGAGACCCACCT |
| SOS2-F | ATTGAGGCTGTAGCGAAC |
| SOS2-R | GGTATTCCTTCTGTTGCC |
| Actin2-F | TCGCTGACCGTATGAGCAAAG |
| Actin2-R | TGTGAACGATTCCTGGACCTG |
